# Polygenic risk score analysis revealed shared genetic background in attention deficit hyperactivity disorder and narcolepsy

**DOI:** 10.1038/s41398-020-00971-7

**Published:** 2020-08-17

**Authors:** Nagahide Takahashi, Tomoko Nishimura, Taeko Harada, Akemi Okumura, Damee Choi, Toshiki Iwabuchi, Hitoshi Kuwabara, Shu Takagai, Yoko Nomura, Jeffrey. H. Newcorn, Nori Takei, Kenji J. Tsuchiya

**Affiliations:** 1 Research Center for Child Mental Development, Hamamatsu University School of Medicine, Hamamatsu, Japan; 2 United Graduate School of Child Development, Osaka University, Kanazawa University, Hamamatsu University School of Medicine, Chiba University and University of Fukui, Osaka/Kanazawa/Hamamatsu/Chiba/Fukui, Japan; 3 Department of Psychiatry, Hamamatsu University School of Medicine, Hamamatsu, Japan; 4 Department of Child and Adolescent Psychiatry, Hamamatsu University School of Medicine, Hamamatsu, Japan; 5 Queens College and Graduate Center, City University of New York, New York, NY USA; 6 Department of Psychiatry, Icahn School of Medicine at Mount Sinai, New York, NY USA; 7 Institute of Psychiatry, Psychology & Neuroscience, King’s College London, London, UK; 8 Present Address: Department of Child and Adolescent Psychiatry, Nagoya University Graduate School of Medicine, Nagoya, Japan

**Keywords:** Clinical genetics, ADHD

## Abstract

Attention deficit hyperactive disorder (ADHD) is a highly heritable neurodevelopmental disorder, and excessive daytime sleepiness is frequently observed in ADHD patients. Excessive daytime sleepiness is also a core symptom of narcolepsy and essential hypersomnia (EHS), which are also heritable conditions. Psychostimulants are effective for the symptomatic control of ADHD (primary recommended intervention) and the two sleep disorders (frequent off-label use). However, the common biological mechanism for these disorders has not been well understood. Using a previously collected genome-wide association study of narcolepsy and EHS, we calculated polygenic risk scores (PRS) for each individual. We investigated a possible genetic association between ADHD and narcolepsy traits in the Hamamatsu Birth Cohort for mothers and children (HBC study) (*n* = 876). Gene-set enrichment analyses were used to identify common pathways underlying these disorders. Narcolepsy PRS were significantly associated with ADHD traits both in the hyperactivity domain (e.g., *P*-value threshold < 0.05, β [SE], 5.815 [1.774]; *P* = 0.002) and inattention domain (e.g., *P*-value threshold < 0.05, β [SE], 5.734 [1.761]; *P* = 0.004). However, EHS PRS was not significantly associated with either domain of ADHD traits. Gene-set enrichment analyses revealed that pathways related to dopaminergic signaling, immune systems, iron metabolism, and glial cell function involved in both ADHD and narcolepsy. Findings indicate that ADHD and narcolepsy are genetically related, and there are possible common underlying biological mechanisms for this relationship. Future studies replicating these findings would be warranted to elucidate the genetic vulnerability for daytime sleepiness in individuals with ADHD.

## Introduction

Attention deficit hyperactive disorder (ADHD) is a common neurodevelopmental disorder with onset occurring in childhood. Genetic factors play an important role in the development of ADHD, with a heritability of up to 75%^1^. Both rare and common genetic variants have been associated with ADHD. Recent genome-wide association studies (GWAS) have identified several genomic regions associated with ADHD, with a modest contribution of common variants to its development^2^.

Recently, based on a polygenic model for complex disorders, polygenic risk scores (PRS) have been calculated and widely used to explain the genetic liability of individuals for certain diseases or phenotypes, using common genetic variants tested in GWAS^3^. PRS can also be used to examine the genetic association between two different diseases or phenotypes. To date, the association of ADHD PRS has been reported with depressive symptoms^4^, body mass index^5^, substance use disorder^6^, and educational attainment^7^, but no examination has thus far been conducted with sleep disorders. Nevertheless, patients with ADHD frequently show excessive daytime sleepiness (EDS)^8^. Narcolepsy is characterized by EDS with or without cataplexy (sudden and uncontrollable muscle weakness or paralysis triggered by a strong emotion), hypnagogic hallucinations, and sleep paralysis (vivid and dream-like hallucinations and muscle paralysis while falling asleep), and disturbed nocturnal sleep. The prevalence of ADHD in individuals with narcolepsy is (~35%)^9^ higher than in the general population (~5%)^10^. Furthermore, psychostimulants, such as methylphenidate, are effective for controlling symptoms of ADHD (first-line approved treatment) and narcolepsy^11^ (frequently used off-line with good effects). However, the nature of the relationship between the two conditions and the possibility that underlying biological mechanisms connect the two have not been elucidated yet.

One recent study conducted in Japan demonstrated that the relative risk of narcolepsy in affected first-degree family members is 10- to 40-fold higher than in the general population, suggesting that genetic factors play an important role in this disease^12^. Of note, the HLA DQB1*15:01 and DQB1*06:02 genes have been shown to be associated with narcolepsy, and additional genes may be involved^13^. Another recent GWAS study, using Japanese individuals, identified some genomic loci related to narcolepsy outside of the major histocompatibility complex (MHC) region, such as *CPT1B*, *TCRA*, and *P2RY11*^12^. Capitalizing on the much higher prevalence of narcolepsy in the Japanese population (0.16–0.18%)^14^ compared to the Caucasian population (0.02–0.06%)^15^, and using the previous Japanese GWAS study of narcolepsy as a discovery cohort, we investigated a possible genetic association between ADHD traits and narcolepsy in our birth cohort sample from the HBC study. We also conducted a polygenic risk analysis of ADHD traits using a summary from the previous GWAS study of essential hypersomnia (EHS), another highly heritable sleep disorder^16^, conducted in a Japanese population^17^. They identified genome-wide significance in single nucleotide polymorphism (SNP) on the *CRAT* gene located in 9q34.11^17^. Although there has been no direct evidence showing the high-concordance rate in twins and familial accumulation of EHS and narcolepsy, it has been reported that these disorders share several susceptibility genes^16^, and ~40% of the heritability of EHS was explained by the genetic background of narcolepsy^12^.

Furthermore, in order to learn more about the common biological mechanisms underlying ADHD and narcolepsy, a gene-set enrichment analysis was performed.

## Methods

### Participants

The discovery cohort for narcolepsy consisted of 409 patients with narcolepsy-cataplexy (narcolepsy type 1: NT1) and 1562 healthy controls. The discovery cohort for EHS was 125 EHS patients without HLA-DQB1*06:02 and 562 controls without HLA-DQB1*06:02. Details are available on the NBDC Human Database (https://biosciencedbc.jp/en/). The target population from the HBC study included infants (*n* = 876) born between December 2007 and June 2011. Recruitment procedures are fully described elsewhere^18^. The study procedures were approved by the Hamamatsu University School of Medicine and the University Hospital Ethics Committee (research ID:17-037 and 19-145). Written informed consent was obtained from each caregiver for his/her infant’s participation. Individuals with intellectual disability (FSIQ < 80, *n* = 66), measured by Wechsler Preschool and Primary Scale of Intelligence (WPPSI), were excluded from the analysis. Also, individuals with parents of non-Japanese descent (*n* = 8) were removed from the study to minimize the effect of population stratification. This study followed the STrengthening the Reporting of OBservational studies in Epidemiology (STROBE) reporting guideline.

### Measurement

ADHD traits were ascertained by interview at age 8 using the Japanese version of the ADHD-RS, previously confirmed for its validity and reliability^19^. ADHD-RS consists of two subscales: inattention (nine items) and hyperactivity-impulsivity (nine items). Each item was rated from 0 (never or rarely) to 3 (very often). The sum of raw scores for inattention, hyperactivity-impulsivity domains, and total scores were converted to percentile scores using the scoring procedure provided in the manufacture’s manual^20^. Full-scale IQ was measured using the Japanese version of the WPPSI, a reliable and child-friendly measure of cognitive development, administered at age 4. All psychological evaluations were conducted by trained psychologists.

### Genotyping, quality control, and imputation

Genotyping was conducted using Japonica array, designed specifically for 650,000 SNP for a Japanese population^21^. In brief, the quality controls for retaining SNPs and individuals were as follows: missing data for SNP < 0.02, Pi-hat calculated by pairwise identify-by-descent (IBD) analysis <0.2, SNP Hardy Weinberg equilibrium of *p* > 10^−6^, and minor allele frequency > 0.01. Seventy-six individuals were removed from the analysis as “related individuals” by IBD analysis. Genotyping imputation was performed using BEAGLE 5.0 to the 1000 Genome Project reference panel phase 3 of the Japanese population^22^. SNPs with an imputation INFO score <0.8 were excluded. We also excluded SNPs located within the MHC region because of high linkage equilibrium in this region. The number of SNPs analyzed for PRS was 5,606,655.

### PRS analysis

We used PRSice-2 to generate PRS, according to the developers’ protocol^23^. The summary GWAS data used to determine the PRS for narcolepsy and EHS was obtained from the NBDC Human Database (https://humandbs.biosciencedbc.jp/en/). To account for population stratification, we included four principle components (PCs). The PCs were calculated based on the pruned data with PLINK 1.9^24^. The criteria for SNP clumping was pairwise LD *r*^2^ < 0.1 within the 1 Mb window. PRSs were calculated with different *P*-value thresholds at 0.05, 0.05, 0.1, 0.5, and 1. Standardized PRS scores (mean = 0 and standard deviation = 1) were used for the analyses.

### Gene-set enrichment analysis

Gene-set enrichment analyses were conducted using PRSet implemented in PRSice-2 software^23^. Gene-sets collections were obtained from the MSigDB database, and GO gene sets (c5) were used for the analyses (http://software.broadinstitute.org/gsea/msigdb/index.jsp). A total GO gene sets used for analysis was 7350. The *P*-value threshold for PRSet was set at 1 since gene-set PRSs containing a small portion of SNPs may be unrepresentative of the whole gene-sets^25^. The obtained *P*-value was corrected for 10,000 permutation tests, and the significance was set at 0.05.

### Genetic correlation analysis

Genetic correlations (r_g_) between narcolepsy and EHS were calculated using LD (linkage disequilibrium) Score regression^26^. Pre-computed LD scores for the East Asian population from the 1000 Genomes Project were used in the analysis (LD scores available on https://github.com/bulik/ldsc) and the summary statistics from the NBDC Human Database was used. Genetic correlation between ADHD and narcolepsy was analyzed using the recent large ADHD GWAS study^2^. Since the difference in the ethnic population between ADHD and narcolepsy GWAS study, we utilized the “Popcorn” program, which can calculate trans-ethnic genetic correlation^27^.

## Results

### PRS analysis

**Fig. 1:**
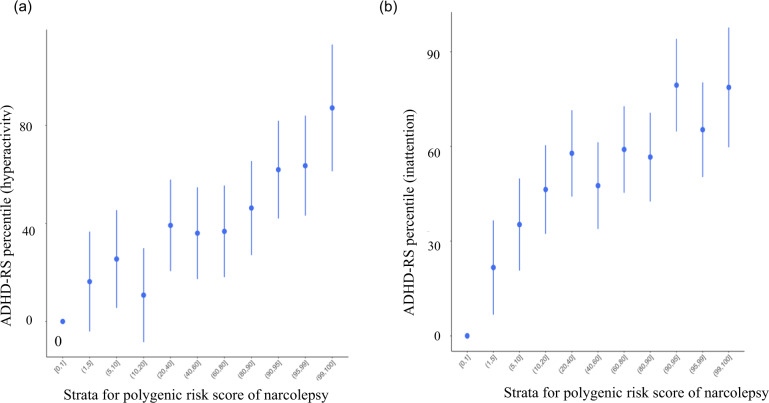
Quantile plot of Narcolepsy PRS and ADHD traits. **a** Quantile plot of Narcolepsy PRS and ADHD traits (hyperactivity). This figure provides the percentile of ADHD-RS hyperactivity score against the strata of polygenic risk score for narcolepsy after the cohort was divided into 100 groups. The error bars mean standard deviation. **b** Quantile plot of Narcolepsy PRS and ADHD traits (inattention). This figure provides the percentile of ADHD-RS inattention score against the strata of polygenic risk score for narcolepsy after the cohort was divided into 100 groups. The error bar means standard deviation. ADHD attention deficit hyperactivity disorder, PRS polygenic risk score.

Participant characteristics are summarized in Table 1. A total of 726 participants (373 males, 353 females) were analyzed. The narcolepsy PRS was significantly associated with ADHD traits at various *P*-value thresholds either in the hyperactivity (e.g., *P*-value threshold < 0.05, *β* [SE], 5.815 [1.774]; *P* = 0.002), inattentive domains (e.g., *P*-value threshold < 0.05, *β* [SE], 5.734 [1.761]; *P* = 0.004) and total score (e.g., *P*-value threshold < 0.05, *β* [SE], 6.496 [1.676]; *P* = 0.001) (Table 2, Fig. 1a, b, Supplemental figure). EHS PRS was not associated with ADHD traits at any *P*-value threshold analyzed (e.g., *P*-value threshold < 0.05), in the hyperactivity domain (*β* [SE], 2.206 [1.726]; *P* = 0.2088) and inattentive domain (*β* [SE], 2.025 [1.715]; *P* = 0.3753); total score (*β* [SE], 1.903 [1.649]; *P* = 0.4174) (Table 3).

**Table 1:** Sample characteristics of children and their parents in the study.

	*n* (%)
Gender	
Male	373 (51.3)
Female	353 (48.7)
Ethnicity	
Japanese	726 (100)
Small for gestational age	
<10th percentile	43 (5.9)
10th–100th percentile	683 (94.1)
Paternal education	
<12 years	62 (8.5)
12 years and longer	664 (91.5)
Maternal education	
<12 years	38 (5.3)
12 years and longer	684 (94.3)
	Mean (SD)
Birthweight (g)	2940.5 (438.8)
Gestational age at birth (weeks)	38.9 (1.6)
Paternal age at birth (years)	33.2 (5.8)
Maternal age at birth (years)	31.5 (5.1)
Household income (million JPY)	6.0 (2.8)
ADHD-RS percentile	
Hyperactivity	52.8 (25.8)
Inattention	50.4 (26.4)
Total	52.3 (29.3)
IQ (WPPSI:FSIQ)	103.3 (17.3)

*SD* standard deviation, *JPY* Japanese Yen, *ADHD-RS* attention deficit hyperactivity disorder rating scale, *IQ* intelligent quotient, *WPPSI* Wechsler Preschool and Primary Scale of Intelligence, *FSIQ* Full Scale IQ.

**Table 2:** Association of narcolepsy PRS and ADHD traits.

ADHD-RS items	*P*-value threshold	Number of SNPs	*R*^2^	Beta	SE	*P*-value^a^
Hyperactivity	0.01	2286	0.011	2.737	1.857	0.146
	0.05	8854	0.078	5.815	1.774	0.002*
	0.1	15124	0.062	4.907	1.795	0.013*
	0.5	46558	0.050	4.07	1.838	0.033*
	1	63954	0.046	3.668	1.805	0.041*
Inattention	0.01	2286	0.011	2.753	1.8437	0.227
	0.05	8854	0.095	5.734	1.761	0.004*
	0.1	15124	0.070	4.167	1.792	0.062
	0.5	46558	0.063	3.627	1.83	0.102
	1	63954	0.060	3.312	1.795	0.105
Total score	0.01	2286	0.018	3.357	1.767	0.112
	0.05	8854	0.110	6.496	1.676	0.001*
	0.1	15124	0.087	5.378	1.703	0.008*
	0.5	46558	0.063	3.893	1.755	0.066
	1	63954	0.059	3.668	1.723	0.079

*PRS* polygenic risk score, *ADHD* attention deficit hyperactivity disorder, *ADHD-RS* attention deficit hyperactivity disorder rating scale, *SNP* single nucleotide polymorphism, *SE* standard error.

^a^*P*-values were corrected using 10,000 permutation tests. * Statistically significant after multiple testing correction.

**Table 3:** Association of essential hypersomnia PRS and ADHD traits.

ADHD-RS items	*P*-value threshold	Number of SNP	*R*^2^	Beta	SE	*P*-value^a^
Hyperactivity	0.01	1839	0.026	0.788	1.900	0.6732
	0.05	7501	0.034	2.206	1.726	0.2088
	0.1	13323	0.040	2.955	1.760	0.1021
	0.5	43288	0.032	2.094	1.772	0.2421
	1	60966	0.031	1.913	1.756	0.2786
Inattention	0.01	1839	0.045	1.053	1.883	0.7773
	0.05	7501	0.050	2.025	1.715	0.3753
	0.1	13323	0.052	2.249	1.753	0.3218
	0.5	43288	0.049	1.851	1.760	0.4437
	1	60966	0.049	1.749	1.744	0.4793
Total score	0.01	1839	0.039	0.665	1.812	0.9265
	0.05	7501	0.045	1.903	1.649	0.4174
	0.1	13323	0.052	2.73	1.682	0.1983
	0.5	43288	0.047	2.228	1.690	0.3234
	1	60966	0.048	2.285	1.674	0.2977

*PRS* polygenic risk score, *ADHD* attention deficit hyperactivity disorder, *ADHD-RS* attention deficit hyperactivity disorder rating scale, *SNP* single nucleotide polymorphism, *SE* standard error.

^a^*P*-values were corrected for multiple testing using 10,000 permutation tests.

### Gene-set enrichment analysis

Gene-set enrichment analyses found that several gene-sets, such as “dopaminergic neuron differentiation” (GO: 0071542; *β* [SE], −5.680 [1.872]; *P* = 0.0034) and “interleukin 6 production” (GO: 0032635; *β* [SE], 6.007 [1.834]; *P* = 0.0006), were associated with hyperactivity traits and narcolepsy (Table 4), while gene-sets, such as “iron coordination entity transport” (GO: 190167; *β* [SE], −6.686 [1.795]; *P* = 0.0002) and “positive regulation of glial cell differentiation” (GO: 0045687; *β* [SE], 5.457 [1.808]; *P* = 0.00350) were significantly associated with both inattention traits and narcolepsy (Table 5).

**Table 4:** Gene-sets significantly enriched for hyperactivity traits and narcolepsy.

Gene-set	*R*^2^	Beta	SE	Number of SNPs	*P*-value^a^
Apoptotic process	0.102	−6.778	1.691	4379	0.0003
Transition metal ion transport	0.082	−5.986	1.759	267	0.0006
Regulation of neurotransmitter levels	0.088	−6.726	1.863	1133	0.0007
Interleukin 6 production	0.078	6.007	1.834	265	0.0011
Heme transport	0.075	−5.865	1.835	26	0.0013
Phosphate ion transmembrane transport	0.078	−6.212	1.894	20	0.0014
Negative regulation of tumor necrosis factor secretion	0.074	−5.930	1.888	11	0.0018
Negative regulation of bone development	0.072	5.599	1.812	26	0.0023
Iron ion transport	0.070	−5.598	1.865	127	0.0027
Positive regulation of reactive oxygen species metabolic process	0.070	−5.342	1.776	102	0.0027
Cerebellar cortex development	0.073	−6.279	2.008	143	0.0028
Dopaminergic neuron differentiation	0.071	−5.680	1.872	68	0.0034
Signal release from synapse	0.076	−5.984	1.862	700	0.0034
Midbrain development	0.068	−5.539	1.892	162	0.0037
Regulation of nitric oxide mediated signal transduction	0.067	−5.417	1.864	38	0.0037
Cellular response to oxidative stress	0.074	−6.202	1.961	650	0.0039
Interferon alpha production	0.066	5.216	1.814	48	0.0042
Positive regulation of tumor necrosis factor superfamily Cytokine production	0.066	5.052	1.762	157	0.0044
Positive regulation of interleukin 12 production	0.066	5.228	1.816	79	0.0044
Positive regulation of interleukin 13 production	0.067	5.171	1.791	26	0.0047

*SNP* single nucleotide polymorphism, *SE* standard error.

^a^*P*-values were corrected for 10,000 permutation tests.

**Table 5:** Gene-sets significantly enriched for inattention traits and narcolepsy.

Gene-set	*R*^2^	Beta	SE	Number of SNPs	*P* value^a^
Heme transport	0.125	−7.488	1.789	260	0.0001
Iron coordination entity transport	0.109	−6.686	1.795	29	0.0002
Negative regulation of cation channel activity	0.096	6.190	1.873	122	0.0008
Regulation of chromatin binding	0.097	−6.327	1.896	49	0.0009
Regulation of presynapse organization	0.091	5.510	1.765	198	0.0010
Postsynaptic specialization organization	0.096	5.917	1.791	302	0.0014
Transition metal ion transport	0.090	−5.468	1.755	267	0.0023
Positive regulation of glial cell differentiation	0.088	5.457	1.808	101	0.0035
Epithelial mesenchymal cell signaling	0.089	5.408	1.774	4	0.0035
Postsynaptic specialization assembly	0.085	5.098	1.745	229	0.0035
Vitamin d metabolic process	0.083	−5.228	1.825	54	0.0040
mRNA cleavage involved in mRNA processing	0.081	−4.676	1.699	13	0.0058
Positive regulation of oligodendrocyte differentiation	0.080	4.848	1.785	62	0.0062
Regulation of oligodendrocyte differentiation	0.081	5.027	1.807	91	0.0066
Formation of cytoplasmic translation initiation complex	0.078	−4.867	1.828	15	0.0070
Radial glial cell differentiation	0.078	4.809	1.816	29	0.0072
Response to corticotropin releasing hormone	0.080	−5.403	1.987	9	0.0075
Multicellular organismal movement	0.080	5.452	1.994	142	0.0078
Transforming growth factor beta secretion	0.077	4.421	1.689	12	0.0083
Synaptic membrane adhesion	0.078	4.795	1.800	245	0.0087

*SNP* single nucleotide polymorphism, *SE* standard error.

^a^P-values were corrected for 10,000 permutation tests.

Exploratory analysis using the Comparative Toxicogenomics Database (http://ctdbase.org) indicated that several compounds, including N-acetylcysteine, baicalein, and fatty acids, were modulators for hyperactivity-related gene ontologies, and dextroamphetamine was a modulator for inattention-related gene-sets.

### Genetic correlation analysis

LD score regression analysis using summary data of previous GWAS studies of narcolepsy and EHS showed that these disorders were positively correlated (r_g_ [SE] = 0.500 [0.062], *P* = 4.42 × 10^−16^). Genetic correlation analysis using summary data of Japanese narcolepsy GWAS and ADHD GWAS conducted by psychiatric genomics consortium showed that these disorders were also positively correlated (r_g_ [SE] = 0.165 [0.114], *P* = 1.11 × 10^−5^).

## Discussion

To our knowledge, this is the first study that has examined the genetic association of ADHD traits and narcolepsy. We found that the genetic risk for narcolepsy has a significant association with ADHD traits using data from the general population. Specifically, both hyperactivity and inattention traits were significantly associated with narcolepsy PRS, suggesting that these disorders have a close genetic association. Genetic correlation analysis using previous GWAS studies of narcolepsy and ADHD also support the present findings.

Managing EDS among individuals with ADHD is important since EDS is directly related to academic performance and teachers’ ratings of classroom behavior and performance^28^. However, EDS in ADHD has usually been considered the result of delayed sleep phase or poor quality of sleep^29^, meaning that the nature of the relationship and the possibility of common underlying biological determinants have not been investigated. Based on the present findings, clinicians, caregivers, and teachers should be informed that there could be a genetic vulnerability for daytime sleepiness in children and adolescents with ADHD. Demontis et al.^2^ found a significant association between ADHD and insomnia. Their finding is not directly linked to our present findings; however, it is also known that subjects with narcolepsy frequently experience disrupted nighttime sleep, which could exacerbate EDS^30^. Future studies examining the relationship between genetic risks for narcolepsy and the quality of nighttime sleep in the present population is needed.

Interestingly, we identified pathways related to common molecular mechanisms underlying ADHD traits and narcolepsy. Several gene-sets were identified as having an association with ADHD symptoms and narcolepsy in the gene-set enrichment analysis. The results are intriguing and bear further study. Notably, gene-sets relevant to dopaminergic signaling were associated with both disorders, consistent with the recognition that most medications currently used for ADHD, narcolepsy and EHS are thought to increase synaptic dopamine^9^. Immune systems have long been implicated in the mechanism of narcolepsy^31^, but recent evidence suggests that neuroinflammation is involved in the development of ADHD as well^32^. Furthermore, neuronal inflammation has been shown to not only alter levels of monoamine neurotransmitters, including dopamine and noradrenaline^32^ but also to affect the expression of histamine and orexin^33,34^, which are promising targets for novel drug treatments for narcolepsy. In addition, gene-sets relevant to glial cell function and iron transport were found to be associated with both ADHD traits and narcolepsy. In line with these findings, recent genetics and brain imaging studies have suggested that altered myelin and oligodendrocyte related signaling were implicated in both ADHD^2,35^ and narcolepsy^36,37^. Changes in iron homeostasis have been shown to affect dopamine synthesis^38^ and also been proposed as a biological mechanism for ADHD^39,40^. Although there has been no direct evidence of iron deficiency in narcolepsy subjects, restless leg syndrome (RLS) was shown to be more prevalent in subjects with narcolepsy compared to general population^41^. The exact pathophysiology of RLS is still unclear. However, it has been repeatedly reported that RLS is also common in ADHD subjects^42^, and iron homeostasis and dopamine signaling have been implicated in the development of this condition^43^. These gene-set enrichment analyses suggest that ADHD traits and narcolepsy share common biological mechanisms such as dopamine signaling, immune system regulation, glial cell function, and iron homeostasis.

We did not find a significant association between EHS PRS and ADHD traits, although genetic correlation analysis showed that narcolepsy and EHS were genetically correlated. One possible explanation for this is that the sample size of the discovery cohort of EHS (Case = 125, control = 1562) was smaller than that of narcolepsy (Case = 409, control = 1562). Thus the calculated EHS PRS did not have adequate power for detecting association with ADHD traits in this study.

Our exploratory analysis using gene-sets enriched in ADHD traits and narcolepsy suggests that medications currently used for ADHD and narcolepsy, such as the psychostimulants, target common biological pathways underlying both disorders. We also found that N-acetylcysteine, baicalein, and fatty acid could be investigated as novel therapeutic agents for both ADHD and narcolepsy symptoms. Although very preliminary, reports suggest the efficacy of N-acetylcysteine for both hyperactivity and inattention symptoms of ADHD^44,45^. N-acetylcysteine has also been shown to be protective for dopamine neurons^46^. Baicalein is a flavonoid purified from the plant *Scutellaria baicalensis*. A recent study using an animal model of ADHD demonstrated the efficacy of core ADHD symptoms by regulating dopaminergic signaling in the brain^47^. More recently, the reduction of fatty acids in the blood^48^ among children and adolescents with ADHD, and similarly, the efficacy of a high dose of eicosapentaenoic acid (EPA) for ADHD symptoms have been reported^49^. Future clinical trials using both existing and novel compounds for ADHD and narcolepsy are warranted.

### Limitations

There are some limitations to this study. First, we targeted a general population, leveraging our longitudinal birth cohort HBC. The cohort is representative of the general population, and thus caution is needed when applying the findings to clinically diagnosed ADHD patients. Second, the original GWAS study of narcolepsy included individuals only with NT1, but not narcolepsy type 2 (NT2), which lacks cataplexy. It is unknown whether the genetic architectures of NT1 and NT2 are the same. Thus, a future GWAS study using NT2 is needed. Third, the prevalence of narcolepsy is known to be higher in the Japanese than the Caucasian population, and future studies using different ethnic populations are needed to replicate these findings.

## Conclusion

In this study, using PRS analysis, we found that genetic risk factors for narcolepsy contribute to ADHD traits in the general Japanese population, and identified common molecular pathways in these conditions. We hope that this discovery will be replicated and lead to an understanding that EDS in ADHD might have a biological foundation.

## Supplementary information

Supplementary figure
