# Supplementary material for: Polygenic risk score analysis revealed shared genetic background in attention deficit hyperactivity disorder and narcolepsy
Source: Transl Psychiatry. 2020 Aug 17;10:284. doi: 10.1038/s41398-020-00971-7 (PMC7429956; doi:10.1038/s41398-020-00971-7)
Supplement: Supplementary file 1 — Supplementary figure [file 41398_2020_971_MOESM1_ESM.docx]

Supplementary figure

(a) Association between narcolepsy PRS and hyperactivity traits at various P-value thresholds


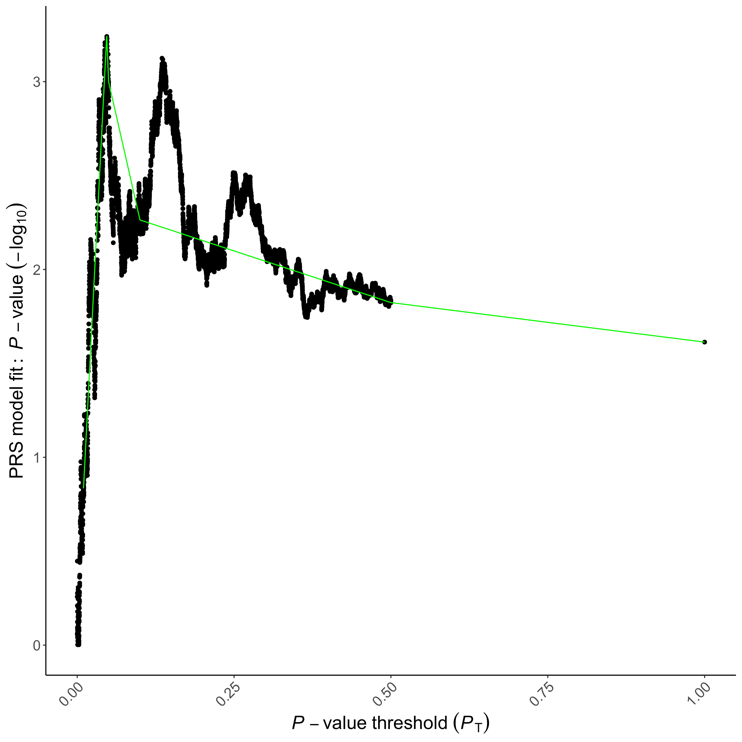


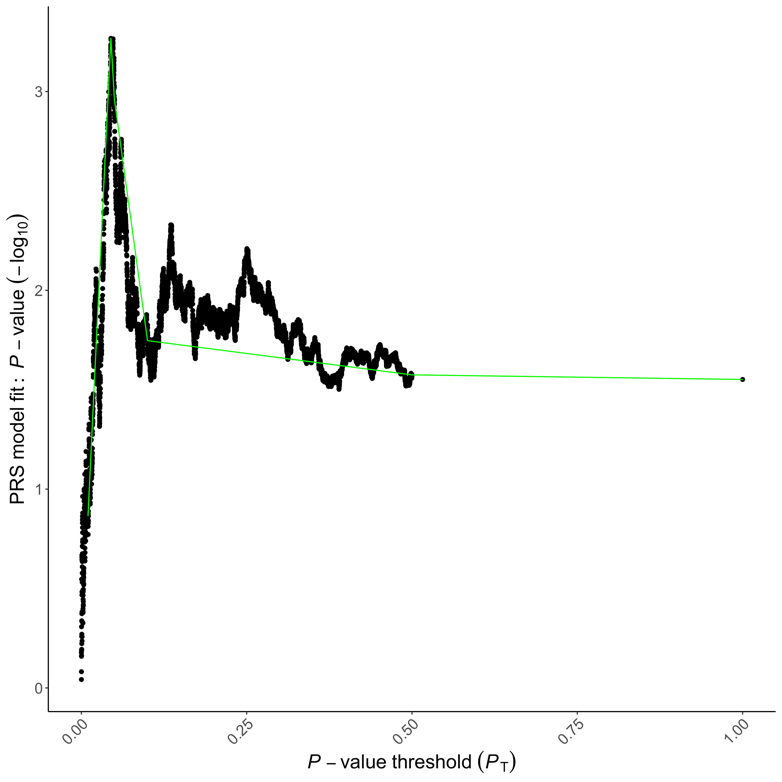
(b) Association between narcolepsy PRS and inattention traits at various P-value thresholds

Abbreviation: PRS, polygenic risk score
